# BFMGHC: a boosted fuzzy manifold granule hypersurface classifier with local topology preservation

**DOI:** 10.3389/fnbot.2026.1899676

**Published:** 2026-07-17

**Authors:** Wei Li, Weiyong Si, Zhisong Liu, Yuping Song

**Affiliations:** 1 School of Information Engineering, Zhejiang Ocean University, Zhoushan, China; 2 School of Computer Science and Electronic Engineering, University of Essex, Colchester, United Kingdom; 3 School of Mathematical Sciences, Xiamen University, Xiamen, China

**Keywords:** brain-inspired computing, finance risk evaluation, hypersurface classifier, local topology structure, manifold

## Abstract

Drawing inspiration from the brain's neurocognitive mechanisms of information chunking and topographic mapping, adaptive decision-making requires neural-grounded architectures that are interpretable and resilient to uncertainty. In this paper, we propose a novel boosted fuzzy manifold granule hypersurface classifier (BFMGHC). The algorithm performs classification at the “information granule" level, realizing an intelligent modeling method that is closer to human cognition, more interpretable, and more accommodating of uncertainty. The classifier mainly consists of three main parts: (1) A manifold-based measurement method for samples that preserves local topological structure is designed, echoing the topographic representations in neural dynamics. Based on this, a global optimization clustering algorithm is proposed and integrated with the Dask framework to achieve scalable hierarchical parallel granulation from raw inputs to high-level semantic granules. (2) In the fuzzy manifold granule space, a measurement method and a hypersurface classifier are constructed, utilizing a particle swarm optimization method for parameter solving. (3) To improve interpretability, weights are assigned to different granules and base classifiers, resembling bio-inspired neuromodulation to ensure stable behavior. The proposed BFMGHC was verified on three financial risk assessment datasets in the UCI Machine Learning Repository (Default of Credit Card Clients, Bank Marketing, and German Credit Data) and achieved superior performance.

## 1 Introduction

Traditional machine learning classifiers often operate as black boxes, lacking the cognitive plausibility required for safe adaptation and human-interpretable behavior in complex control systems. In contrast, the human brain excels at processing highly uncertain environmental stimuli by abstracting continuous sensory inputs into discrete, high-level semantic symbols—a process known as cognitive chunking or granulation. Furthermore, neurophysiological evidence suggests that the cerebral cortex represents structured knowledge using low-dimensional manifold topologies (e.g., grid cell networks in the hippocampal formation). To bridge the gap between abstract mathematical classification and brain-inspired cognitive modeling, this paper proposes BFMGHC. By embedding local topological preservation and fuzzy granulation, our model mimics the neural principles of cross-disciplinary cognitive efficiency and provides an interpretable, robust decision-making architecture suitable for hybrid neuro-mechanical or adaptive control environments. Granular computing (GrC) was first proposed by Zadeh (1979, 1997). It is a powerful tool for simulating human thinking and solving structured solution patterns, as well as processing uncertain information, for large-scale complex problems. It is not only a natural characteristic of human cognition, but also an inherent requirement of many intelligent analysis tasks (Miao et al., 2016). In existing studies, researchers have developed various granular computing models from different research perspectives, including the fuzzy set theory, rough set theory, cloud, quotient space theory, and three-branch decision theory.

Rough set theory (Pawlak, 1982) is one of the more mature theories in the theoretical framework of granular computing, with a broader range of applications. It is an effective theory and tool for processing uncertain information and discovering knowledge. It can mine causal relationships within data itself from different information systems through conceptual approximation, without the need for prior information, and extract valuable information contained therein. Classical Pawlak rough sets form equivalence classes on the domain based on equivalence relations (i.e., indistinguishable relations) between objects, thereby achieving approximation and approximation of different target concepts. In many practical decision-making problems, decision information may be a combination of information from multiple sources and with diverse attribute types. For example, clinical diagnosis and treatment information may include data obtained from various sources, such as the patient's chief complaint, the doctor's assessment of the patient's vital signs, and auxiliary examination information gathered through instrument and equipment inspections. At the same time, these data may include multiple attribute types such as symbolic values, real values, language values, and images. However, the classical Pawlak rough set is only suitable for processing discrete data. If it is used directly to process other types of information, it may inevitably lead to information loss due to the discretization of data. Given this, scholars have developed new rough set models by expanding binary relations to accommodate the decision-making needs of diverse data types in practice. Fuzzy rough sets (Dubois and Prade, 1990) replace the indistinguishable relationship between objects with fuzzy similarity relations, which not only retains the advantages of classical rough sets in processing discrete values, but also provides a new theory for processing decision problems with real-valued attributes. Hu et al. (2008) introduced neighborhood relations into rough sets, improving the feature evaluation capability and classification performance of heterogeneous data. By defining mixed indistinguishable relations, Sun et al. (2014) defined a mixed attribute information fuzzy rough set based on indistinguishable mixed ties, providing a new method for rough approximation of fuzzy concepts based on mixed attribute information. Zhang et al. (2014) constructed a composite rough set model by integrating different binary relations (such as equivalence relations, neighborhood relations, and tolerance relations), which is used to deal with decision problems in composite information systems containing multiple data types. Hu et al. (2011) introduced the kernel method in machine learning into rough sets and constructed kernel fuzzy rough sets. The kernel-based rough set model avoids data type conversions and simplifies the classification task by mapping data to a high-dimensional, linearly separable space. Subsequently, scholars conducted further research on the basis of kernel fuzzy rough sets. Zeng et al. (2015) constructed a hybrid distance based on value difference measurement in a hybrid information system, and designed a novel fuzzy rough set approach in combination with a Gaussian kernel function. Hu et al. (2017) introduced multi-kernel learning into fuzzy rough set, defined the binary relationship of multimodal data by combing various kernel mappings, and constructed multi-kernel fuzzy rough set on this basis. Yang et al. (2022a) innovatively merged Student-t kernel fuzzy divergence with the fuzzy rough set paradigm, resulting in a refined model known as the Student-t kernel fuzzy rough set.

As mentioned above, in practice, decision information may be multi-source information from different information sources in addition to being of various types. Based on the data characteristics from various information sources, using different binary relationships to characterize decision objects based on the distinct characteristics of each information source's data may be more in line with actual decision-making scenarios. The rough set framework incorporating multigranularity proposed by Qian et al. uses a multigranularity knowledge representation method to integrate multiple binary relationships between objects based on multiple sources of information or granularity levels in the information merging process, which can more comprehensively express the relationship between objects and provide global knowledge guidance for knowledge discovery based on data from different data sources. In recent studies, researchers have used multigranularity rough set theory to study the information fusion problem from multiple data sources. Che et al. employed evidence-theoretical analysis with information entropy to investigate the synthesis of information from diverse sources problem in multisource information systems within a multigranularity framework. Guo et al. (2020) introduced a granular weighting technique grounded in decision trees from a machine learning standpoint, and developed a weighted multigranular rough set model to tackle the fusion of multisource information. Aiming at multi-source dynamic information, Zhang et al. (2023) proposed a multigranular fusion approach grounded in matrix theory. Real-world decision problems may also involve data with multilevel hierarchical structures. Wu and Leung (2011) first proposed a multiscale information system and the corresponding rough approximation. Multiscale data systems embody the basic idea of granular computing to address multilevel and multiview complex problems and provide a new perspective and approach for processing multi-level structured data (Wu et al., 2017; Huang and Li, 2021). The aforementioned research on rough set theory not only enhances its fundamental framework but also offers novel perspectives for exploring complex information fusion and knowledge discovery. This paper also draws on the theoretical basis for studying the problem of information fusion in clinical diagnosis and treatment data.

Yao (2012) first proposed the three-branch decision-making method, which is another essential theoretical method in the field of granular computing. In reality, due to the incompleteness and imprecision of decision information, decision makers can only delay decision-making when the existing decision information cannot determine the object of rejection or acceptance of the decision, and wait for the information to be supplemented and improved before making a judgment, to reduce the risk of decision-making and enhance the accuracy of decision-making. In three-branch decision-making, the positive domain, boundary domain, and negative domain of a rough set are mapped to three decision behaviors: acceptance, non-commitment, and rejection. It provides an interpretation rule based on decision semantics for these three areas. This process effectively simulates the thought process of human beings in solving practical problems. In 2018, Yao (2018) investigated the connection between three-branch decision-making and granular computing based on the traditional narrow three-branch framework, proposed the “Trisecting-Acting-Outcome” (TAO) model from a fresh perspective, and further clarified the principles and significance of the three-branch decision-making concept. Since the three-branch decision-making idea was first introduced, numerous scholars have conducted extensive research on this approach from both theoretical and practical perspectives. In terms of theory and method, combined with other granular computing models and group decision-making and machine learning theories, three-branch decision space (Hu, 2014), three-branch group decision (Liang et al., 2015), three-branch concept analysis (Li et al., 2017; Huang et al., 2017), three-branch classification (Liu, 2021), three-branch recommendation (Zhang and Min, 2016), three-branch clustering (Yu et al., 2016). Other theories and methods have been developed over time. In terms of application, the three-branch decision has been successfully applied to various fields, including emergency management (Sun et al., 2018), medical decision-making (Wang et al., 2022), conflict analysis (Sun et al., 2020; Yao, 2019), and image processing (Li et al., 2016), among others. Given the multi-angle and multi-dimensional problems of decision information in actual management decision-making, some scholars have theoretically expanded the theory of three-branch decision from the perspective of multigranularity, achieving rich research results. Qian et al. (2020) proposed a sequential decision model under a multigranularity framework. On this basis, they further integrated both aspects to develop a generalized hierarchical multigranularity sequential three-branch decision model (Qian et al., 2022). Huang et al. (2020) studied the multigranularity three-branch decision problem of multi-scale information. Zhang et al. (2020) investigated the multigranularity three-branch decision-making process for hesitant fuzzy language information under dual domains. Xue et al. (2020) constructed a multigranularity three-branch decision based on supporting intuitionistic fuzzy sets. Yang et al. (2022b) constructed a multi-layer granular structure with spatiotemporal characteristics using data that increases over time and feature spaces with high fitting degree, and studied the spatiotemporal multigranularity three-branch granular computing model for dynamic data. The three-branch clustering method (Yu et al., 2016) integrates the principles of three-branch decision-making into the domain of cluster analysis. Based on the positive, boundary, and negative domains in three-branch decision theory, the method defines the corresponding core, edge, and trivial regions within a cluster. In contrast to clustering methods based on two-branch decisions that represent a cluster with a single set, the three-branch clustering approach employs two sets—the core and edge regions—to represent each class. Hence, three-branch clustering offers a more refined representation of the inherent uncertainty associated with clustering. Three-branch clustering offers an effective tool for examining uncertainty in clustering. There are many studies on three-branch clustering. By introducing three-branch weights, Zhang (2019) developed a C-means-based three-branch clustering algorithm to enhance the modeling of uncertainty in clustering. Yu et al. (2020) designed an adaptive clustering threshold three-branch clustering algorithm based on gravity search. A variance-based three-branch clustering approach was introduced by Afridi et al. (2020), in which an optimization criterion is used to determine the threshold within each cluster. Yu et al. (2019) developed an integrated three-branch clustering method within the Spark framework to address the clustering challenges posed by large-scale data. To mitigate the issue of clustering label error propagation, they further proposed a density peak-based three-branch clustering algorithm grounded in evidence theory (Yu et al., 2021). Li et al. (2024) designed a boosted stochastic fuzzy granular hypersurface classifier to resolve the classification issue of numerical data and non-numerical data from the standpoint of granular computing. In a related but distinct line of research, Huang et al. (2025) explored unpaired image-text matching, a novel scenario in which paired image-text data is assumed to be unavailable during model training. Tu et al. (2025) proposed Attribute-missing graph learning. Mahner et al. (2025) presented an interpretability framework that compares how humans and deep neural networks process images. These pushed the development of AI. This paper, from the perspective of granular computing, systematically reviews the theoretical developments and practical applications of rough sets, fuzzy rough sets, multigranularity rough sets, and three-way decision-making in handling uncertainty, fusing multisource information, and supporting complex decisions. As one of the most representative models in the granular computing system, the rough set theory has been effectively expanded to adapt to the processing needs of heterogeneous data, multi-type attributes, and high-dimensional complex data. In particular, the successive introduction of fuzzy rough sets, kernel rough sets, and multigranularity rough sets models has provided a solid foundation for knowledge discovery and feature expression in complex information systems. At the same time, as an essential granular computing paradigm that simulates the three types of human behavior patterns of “acceptance-delay-rejection”, the three-way decision-making has not only been continuously enriched in theoretical modeling, but also widely used in many practical fields such as medical care, emergency response, recommendation, clustering, etc., showing a powerful ability to model decision-making behavior in incomplete and uncertain environments. In particular, the research on three-way clustering and multi-granularity three-way decision-making has fully demonstrated the advantages of granular computing in multi-level structured representation and uncertainty modeling. In summary, the current development of granular computing-related theories has evolved from preliminary rough descriptions to the stage of composite modeling that integrates multi-perspective, multi-scale, and multi-type information, which not only expands the expressive power of the model but also provides a more interpretable, robust, and cognitively aligned technical path for dealing with practical complex problems. The theoretical review and summary of this article lay a theoretical foundation and provide methodological guidance for subsequent research on information fusion modeling and intelligent decision-making in highly complex scenarios, such as clinical diagnosis and treatment.

### 1.1 Motivation

In the context of the increasing popularity of data-driven intelligent decision-making, classification models need to have not only high accuracy, but also be able to effectively deal with uncertainty in data and provide explanatory reasoning close to human cognitive methods. However, most traditional machine learning and deep learning methods rely on sample-based rigid decisions and assume that data obeys the Euclidean structure. When faced with high-dimensional, nonlinear, and noise-perturbed real data, it is often challenging to capture local topological features and address issues such as fuzzy category boundaries and complex sample distributions.

Especially in high-risk practical applications such as financial risk assessment, the model should not only have good generalization capabilities, but also support information abstraction and uncertainty expression at the semantic level to achieve a more robust and explainable decision-making process. However, existing classification methods generally lack a structured modeling mechanism from “data to cognition" and cannot complete effective learning at the level of high-level semantic granules.

To this end, it is urgent to break through the traditional point-level modeling paradigm, build an intelligent classification framework based on “information granules,” aggregate similar samples into fuzzy granules, and use them as basic cognitive units for reasoning and decision-making. If the local topological structure is maintained by manifolds, the expressive power of fuzzy granulars and the integration mechanism of multiple classifiers can be integrated, making it possible to realize a new intelligent classification model with interpretability, robustness, and cognitive alignment characteristics. Based on the aforementioned requirements, this paper proposes a boosted fuzzy manifold granule hypersurface classifier, which comprehensively explores the deep integration of granular computing and intelligent classification, from topological similarity modeling to fuzzy granule space construction and integrated decision-making mechanisms, striving to achieve better performance and stronger cognitive interpretation ability in complex data scenarios.

### 1.2 Contributions

#### 1.2.1 Innovation point 1

Manifold-preserving local topology structure granulation framework (scalable parallel implementation). A similarity measure based on local topology structure graphs and a global optimization clustering algorithm is introduced to address the shortcomings of traditional Euclidean distance on high-dimensional/nonlinear data. Combined with Dask parallel granulation, scalable abstraction from raw data to high-level semantic information granules is achieved, laying the foundation for large-scale uncertain data modeling.

#### 1.2.2 Innovation point 2

Fuzzy manifold granule hypersurface classification model and particle swarm parameter optimization. A hypersurface classifier is constructed in the fuzzy manifold granule space to naturally express the uncertainty of category boundaries using fuzzy metrics; particle swarm optimization is employed to jointly search for classifier parameters, making the model more robust and interpretable in the presence of noise, transition areas, and imbalanced scenarios.

#### 1.2.3 Innovation point 3

Comprehensive decision-making mechanism for weighted integration of multiple fuzzy manifold granule hypersurface classifiers. By setting weights for different fuzzy granules and their base classifiers, an interpretable and controllable integrated decision framework is formed. While maintaining model diversity, the overall prediction performance and generalization ability are improved, making it suitable for complex practical scenarios, such as financial risks.

## 2 Problems

Given a classification system defined as


𝕊=(X,Y,A,V,τ),

where

- $X={\left\{x_{1},x_{2},\ldots ,x_{n}\right\}}^{T}$ is the sample set, with each $x_{i}\in {\mathbb{R}}^{m}$ representing the *m*-dimensional attribute vector of the *i*-th sample;
- *Y* = {*y*_1_, *y*_2_, …, *y*_*n*_} is the set of class labels corresponding to the samples, where each *y*_*i*_ ∈ ℝ denotes the category label of the sample *x*_*i*_;
- *A* = {*a*_1_, *a*_2_, …, *a*_*m*_} is a non-empty finite set of attributes;
- *V* = ⋃_*a*∈*A*_*V*_*a*_ denotes the full set of attribute values, where *V*_*a*_ is the value domain of attribute *a*;
- τ: *X* × *A* → *V* is an information function assigning to each attribute of each sample a corresponding attribute value, i.e.,

$$\forall a\in A,\forall x\in X,\;\tau \left(x,a\right)\in V_{a}.$$

The classification system *S* consists of two parts:

1. **Learning system:** uses existing training data to build a classification model, that is, a learning function
F:X→Y,
  such that *Y* = *F*(*X*);
2. **Prediction system:** applies the learned function *F* to predict the class label *y*_*n*+1_ for a test sample *x*_*n*+1_.

Under the proposed framework, the BFMGHC process is divided into two key stages: firstly, the parameter solution stage responsible for model training, and secondly, the prediction stage applied to classify unseen data. In the parameter solution stage, the training samples are first clustered to obtain the cluster center set of the data; then, the metric relationship between each sample and each cluster center is calculated based on the similarity of the local topological structure to reveal its local nested structure in the feature space. Subsequently, the original data is fuzzy granulated with the help of the Dask distributed computing framework, and the samples are mapped into fuzzy manifold granule vectors with fuzzy uncertainty, and corresponding weights are assigned to each vector. On the set of fuzzy manifold granule vectors, *T* base fuzzy manifold granule hypersurface classifiers {*f*_1_, *f*_2_, ···, *f*_*T*_} with different weights are trained. By weighted linear combination of these classifiers, the final integrated classification model is constructed, that is, the BFMGHC model *F*(*x*) is thus enhanced, which improves both the generalization ability and stability of the classifier.

**Figure 1:**
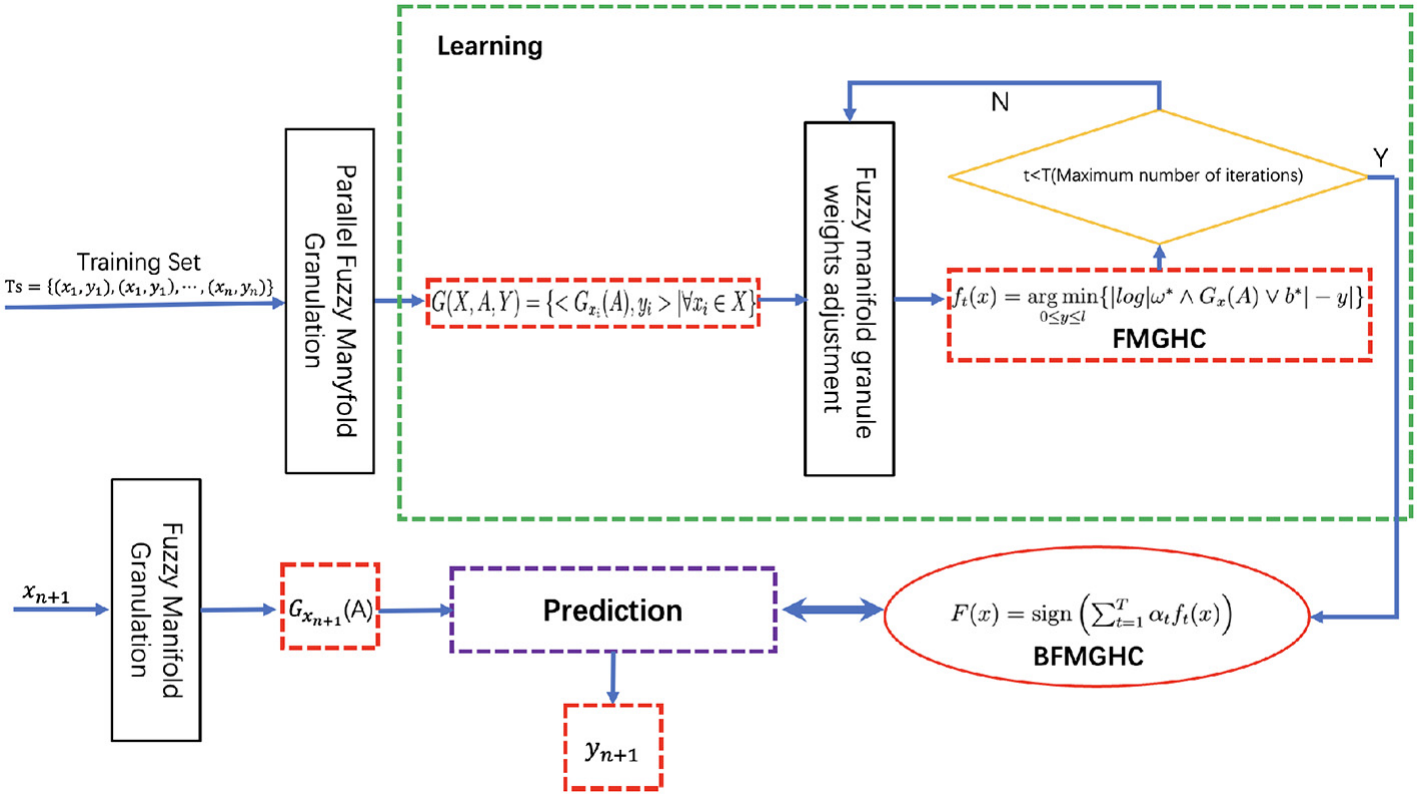
The overflow of BFMGHC.

In the prediction stage, for a sample to be classified, it is firstly subjected to fuzzy manifold granulation consistent with the training stage to obtain its fuzzy manifold granule representation; then, it is input into the trained integrated model *F*(*x*), and its corresponding prediction category is output. The entire classification process incorporates fuzzy embedding and manifold structure modeling while preserving the local sensitivity advantages of granular computing, thereby achieving both interpretability and strong predictive performance. The overall workflow is illustrated in Figure 1.

## 3 From data to fuzzy manifold granules

### 3.1 Global optimization clustering algorithm with local topology structure preservation

In traditional clustering methods, some classical algorithms, such as k-means, necessitate the pre-specification of the number of clusters and the cluster centers to be initialized, and then the final clustering results are obtained by iteratively updating the cluster centers. For the similarity measurement between samples, most methods use the direct calculation of the distance between samples (such as Euclidean distance or Manhattan distance). However, these methods have several obvious defects: Firstly, these algorithms are highly sensitive to the choice of initial cluster centers and the number of clusters. An improper selection of initial centers can cause the algorithm to converge to a local optimum, thereby failing to achieve the global optimal clustering solution. Secondly, many distance measurement methods cannot accurately and objectively measure the similarity between samples in some complex scenarios, especially when facing non-spherical or complex topological data. Traditional distance measurement methods perform poorly.

In order to address the aforementioned issues, this paper proposes a novel Global Optimization Clustering Algorithm with Local Topology Structure Preservation (GOCALTSP). The proposed method is capable of automatically determining both the number of clusters and the cluster centers, while ensuring global optimization. Moreover, it evaluates the similarity between samples by incorporating their local topological structure. Unlike traditional methods, the proposed algorithm does not rely on a predefined number of clusters. Instead, it adaptively selects both the cluster centers and their number to maximize clustering performance, thereby avoiding the drawbacks associated with dependence on initial parameters. By introducing the measure of local topological structure, we can more accurately describe the similarity between samples, especially in high-dimensional complex data, and avoid the limitations of traditional distance measurement methods.

The effect of clustering is usually evaluated by measuring the variance between clusters and the variance within clusters. Ideally, the variance between clusters should be large and the variance within clusters should be small. Only when this condition is met, the clustering result is effective and high-quality. Therefore, we propose the following loss function as an evaluation index for clustering effect:


$$\begin{array}{l} L\left(C,X\right)=log\left(\overset{k}{\underset{i=1}{\sum }}\sigma_{i}^{2}\right)-log\delta^{2}-log\overset{k}{\underset{i=1}{\sum }}\underset{x\in c_{i}}{\sum }\cos\left(c_{i},x\right) \end{array}$$ (1)

Where δ denotes the standard deviation of the cluster centers, σ_*i*_ is the standard deviation of the sample points within the *i*th cluster, *k* represents the total number of clusters, *c*_*i*_ denotes the *i*th cluster center, and cos(*c*_*i*_, *x*) represents the similarity measure between the cluster center and a sample point (e.g., cosine similarity).

The smaller the value of the loss function, the better the clustering performance, indicating a more effective clustering result and a stronger capability of the model to capture intrinsic data structures. We aim to minimize the loss function by continuously adjusting the cluster center to achieve the goal of global optimization.

The optimization process of the algorithm follows the following steps: First, the cluster center is initialized and clustering iterations are performed. Each iteration reduces the value of loss function by updating the cluster center. Specifically, in each iteration, the algorithm computes the standard deviation of the current cluster center, the standard deviation of the samples within each cluster, and the similarity between the samples and the cluster center, and subsequently updates the cluster centers accordingly. The algorithm continues to iterate until the loss function value no longer changes significantly or reaches the predetermined maximum number of iterations.

In each iteration, a new set of cluster centers is generated, and the corresponding loss function value is calculated. To further evaluate the clustering performance, the cluster centers and their associated loss values from each iteration are added to an evaluation set. Once the termination condition is satisfied, the algorithm selects the set of cluster centers with the minimum loss function value from the evaluation set as the final clustering result. This strategy effectively avoids entrapment in local optima and ensures the stability and superiority of the final outcome.

In summary, the proposed random GOCALTSP effectively overcomes the shortcomings of traditional clustering methods in parameter selection and measurement similarity by adaptively selecting cluster centers and numbers and introducing local topological structure metrics, and can obtain more accurate and efficient clustering results in complex data environments.

#### 3.1.1 Metrics that preserve local topology structure

The similarity calculation of samples *x*_*i*_ and *x*_*j*_ does not directly calculate the distance between them in this paper, but forms a local manifold topology map with the nearest *t* neighbors around each of them as the center, and calculates the distance between the manifold topology maps to express the similarity between *x*_*i*_ and *x*_*j*_. Specifically as follows: *x*_*i*_ is a point defined in an *m*-dimensional space (manifold *X*), and its *m* attribute values are ${\left(x_{i1},\cdot \cdot \cdot ,x_{im}\right)}^{T}$. Similarly, *x*_*j*_ is a point defined in an *m*-dimensional space (manifold *X*), and its *m* attribute values are ${\left(x_{j1},\cdot \cdot \cdot ,x_{jm}\right)}^{T}$. *P*_*x*_*i*__ is a (*t*+1) × (*t*+1) represents the local geometry matrix of *x*_*i*_. Here, *P*_*x*_*i*__(*c, d*) = *distance*(*q*_*c*_, *q*_*d*_). *q*_1_ = *x*_*i*_, {*q*_2_, ···, *q*_*t*+1_} is the *t* neighbor of *x*_*i*_. Similarly, *P*_*x*_*j*__ represents a *x*_*j*_ is the local geometric (*t*+1) × (*t*+1) matrix. *x*_*i*_'s *t* nearest neighbors have *t*! permutations, so *P*_*x*_*i*__ has *t*! variables. Let {_*P*_*x*_*i*__}*u*_ denote its *u*th variable. We quantify the distance between *x*_*i*_ and *x*_*j*_ using the following metric:


$$\begin{array}{l} \begin{array}{l} distance\left(P_{x_{i}},P_{x_{j}}\right)=\underset{1\leq u\leq t!}{\min}\\ \;\min\left(distance_{1}\left(u\right),distance_{2}\left(u\right)\right) \end{array} \end{array}$$ (2)

here


$$distance_{1}\left(u\right)=||{\left\{P_{x_{j}}\right\}}_{u}-h_{1}P_{x_{i}}|{|}_{F}$$

$$distance_{2}\left(u\right)=||P_{x_{i}}-h_{2}{\left\{P_{x_{j}}\right\}}_{u}|{|}_{F}$$

$$h_{1}=trace\left(P_{x_{i}}^{T}{\left\{P_{x_{j}}\right\}}_{u}\right)/trace\left(P_{x_{i}}^{T}P_{x_{i}}\right)$$

$$h_{2}=trace({\left\{P_{x_{j}}\right\}}_{u}^{T}P_{x_{i}}/trace({\left\{P_{x_{j}}\right\}}_{u}^{T}{\left\{P_{x_{j}}\right\}}_{u})$$

**Theorem 1**. Given two (*t*+1) × (*t*+1) distance matrices *P*_1_ and *P*_2_,

$h_{2}=trace\left(P_{2}^{T}P_{1}\right)/trace\left(P_{2}^{T}P_{2}\right)$ minimizes ||*P*_1_−*h*_2_*P*_2_||_*F*_, and

$h_{1}=trace\left(P_{1}^{T}P_{2}\right)/trace\left(P_{1}^{T}P_{1}\right)$ minimizes ||*P*_2_−*h*_1_*P*_1_||_*F*_.

Proof:

Find *h*_2_ such that *h*_2_ = *argmin*_*h*_2__||*P*_1_−*h*_2_*P*_2_||_*F*_ Here ||·||_*F*_ represents the Frobenius norm.

It is easy to prove that $||P_{1}-h_{2}P_{2}|{|}_{F}=trace\left(P_{1}^{T}P_{1}\right)-2h_{2}trace\left(P_{2}^{T}P_{1}\right)+h_{2}^{2}trace\left(P_{2}^{T}P_{2}\right)$. Because $trace\left(P_{1}^{T}P_{1}\right)$ is a constant, the minimization problem is equivalent to


$$h_{2}=argmin_{h_{2}}\left\{h_{2}^{2}trace\left(P_{2}^{T}P_{2}\right)-2h_{2}trace\left(P_{2}^{T}P_{1}\right)\right\}$$

Regarding the differentiation of *h*_2_, we get


$$2h_{2}trace\left(P_{2}^{T}P_{2}\right)=2trace\left(P_{2}^{T}P_{1}\right)$$

That is,


$$h_{2}=trace\left(P_{2}^{T}P_{1}\right)/trace\left(P_{2}^{T}P_{2}\right)$$

Similarly,


$$h_{1}=trace\left(P_{1}^{T}P_{2}\right)/trace\left(P_{1}^{T}P_{1}\right)$$

#### 3.1.2 Adaptive clustering algorithm preserving local topology structure

In the previous section, we proposed a metric algorithm for points on two manifolds that preserves local topology structure. Based on this, we will give an adaptive clustering algorithm that preserves local topology structure.

The algorithm is briefly described as follows:

**Step 1:** Remove samples that possess default or invalid eigenvalues.

**Step 2:** Normalize the attribute values of all samples using the Gaussian function.

**Step 3:** Set the maximum iteration count to *MaxIter*, initialize the evaluation set *E* as empty, i.e., *E* ← ∅. The evaluation set stores pairs of cluster center sets and their corresponding loss values. Initialize the iteration counter as *iteration* = 1.

**Step 4:** Initialize the current cluster center set as *C*_*iteration*_ = ∅. Randomly determine *k* as the number of cluster centers. Select one sample *x*_*j*_ at random to serve as the first cluster center, set *t* ← 1, *c*_*t*_ ← *x*_*j*_, and update *C*_*iteration*_ ← *C*_*iteration*_∪{*c*_*t*_}.

**Step 5:** For each sample, compute the minimum distance to the existing cluster centers:


$$s\left(x_{j}\right)=\underset{c_{i}\in C_{iteration}}{\min}\left\{\text{distance}\left(P_{c_{i}},P_{x_{j}}\right)\right\},$$

where distance(·, ·) is defined in Equation 1. Then, calculate the probability of selection as the next cluster center by


$$p\left(x_{j}\right)=\frac{s{\left(x_{j}\right)}^{2}}{\sum_{i=1}^{n}s{\left(x_{i}\right)}^{2}},\;j=1,2,\ldots ,n.$$

**Step 6:** If *x*_*j*_ is chosen, increment the cluster count: *t* ← *t*+1, assign *c*_*t*_ ← *x*_*j*_, and update the cluster center set *C*_*iteration*_ ← *C*_*iteration*_∪{*c*_*t*_}.

**Step 7:** If *t* ≤ *k*, return to **Step 5**; otherwise, proceed to **Step 8**.

**Step 8:** Compute the loss function value *L*(*C*_*iteration*_, *X*) for the current iteration, then update the evaluation set:


$$E\leftarrow E\cup \left\{\left(C_{iteration},L\left(C_{iteration},X\right)\right)\right\}.$$

**Step 9:** Increment the iteration counter:


iteration←iteration+1.

**Step 10:** If either the stopping condition


iteration>MaxIter

is met, or for all *N* ∈ (1, *a*−*b*),


$$∥L\left(C_{N},X\right)-\frac{L\left(C_{a},X\right)+L\left(C_{b},X\right)}{2}{∥}^{2}<\epsilon ,$$

where *b* + *N* < *a* and ϵ is a small positive threshold, then proceed to **Step 11**; otherwise, return to **Step 4**.

**Step 11:** From the evaluation set *E*, select the cluster center set corresponding to the minimal loss function value:


$$C^{*}=\arg\underset{1\leq iteration\leq MaxIter}{\min}L\left(C_{iteration},X\right),$$

$$\;C^{*}=\left\{c_{1},c_{2},\ldots ,c_{\left|C^{*}\right|}\right\},$$

where |·| denotes the cardinality of the set.

The above describes the globally optimized adaptive clustering methodology. The pseudo-code implementation is provided in Algorithm 1.

Algorithm 1 Global optimization clustering algorithm with local topology structure preservation. 
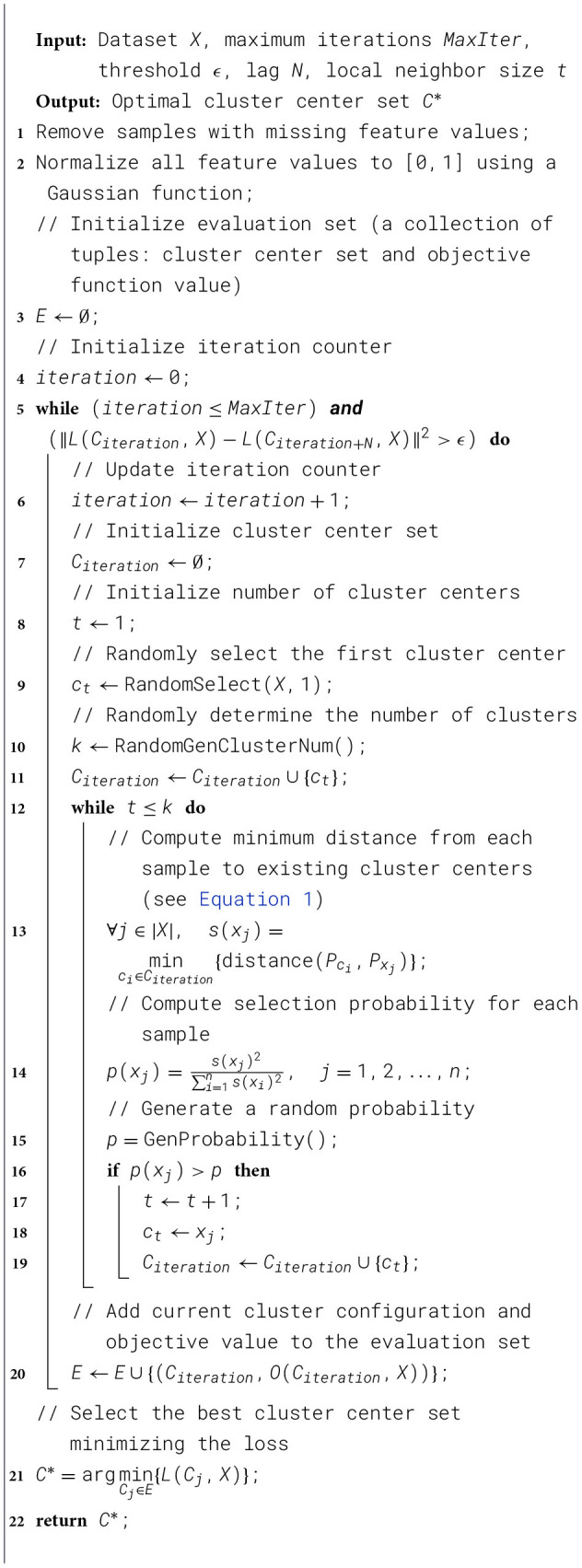

### 3.2 Parallel fuzzy manifold granulation

Classic data granulation methods rely on calculating pairwise similarities in a sequential manner, resulting in a similarity matrix that is unsuitable for parallel processing. We propose a clustering-first-and-parallel granulation method. First, the samples are divided into several subsets. The cluster center set of the sample can be granulated with each subset, which can meet the conditions for parallel execution and greatly improve efficiency. According to the algorithm in Table 1, let the cluster center of the sample be $C^{*}=\left\{c_{1},c_{2},...,c_{\left|C^{*}\right|}\right\}$. For ∀*x*_*i*_ ∈ *X*, ∀*a*_*t*_ ∈ *A* and $\forall c_{j}\in C^{*}$, the similarity between *x*_*i*_ and *c*_*j*_ on *a*_*t*_ can be written as:


$$\begin{array}{l} s_{x_{i},c_{j}}\left(a_{t}\right)={\left(1+exp\left(distance\left(P_{x_{i}}\left(a_{t}\right),P_{c_{j}}\left(a_{t}\right)\right)\right)\right)}^{-1} \end{array}$$ (3)

**Table 1:** Data sets from UCI machine learning repository.

Data set	Number of instances	Number of attributes
Default of credit card clients	30,000	23
Bank marketing	45,211	16
Statlog (German credit data)	1,000	20

Obviously 0 < *s*_*x*_*i*_, *c*_*j*__(*a*_*t*_) < 1. Then a manifold granula induced by *x*_*i*_ and cluster center *c*_*j*_ on the *a*_*t*_ attribute can be written as:


$$\begin{array}{l} g_{x_{i}}\left(a_{t}\right)=s_{x_{i},c_{1}}\left(a_{t}\right)+s_{x_{i},c_{2}}\left(a_{t}\right)+\cdot \cdot \cdot +s_{x_{i},c_{\left|C^{*}\right|}}\left(a_{t}\right) \end{array}$$ (4)

For simplicity, it can also be written as


$$\begin{array}{l} g_{x_{i}}\left(a_{t}\right)=\int_{c_{j}\in C^{*}}s_{x_{i},c_{j}}\left(a_{t}\right) \end{array}$$ (5)

Note that the symbol “∫” in this context represents the union over sets, not the integral. That is, the manifold granula *g*_*x*_*i*__(*a*_*t*_) represents the similarity between the sample *x*_*i*_ and the cluster center set C^*^ on the attribute *a*_*t*_. Its modulus can be written as:


$$\begin{array}{l} |g_{x_{i}}\left(a_{t}\right)|=\overset{\left|C^{*}\right|}{\underset{j=1}{\sum }}s_{x_{i},c_{j}}\left(a_{t}\right) \end{array}$$ (6)

Below we design four operators about manifold granules.

∀*e, f* ∈ *R*, operators ∪ and ∩ can be defined as follows:


$$\begin{array}{l} e\cup f=\lambda ef+\left(1-\lambda \right)\left(e+f-ef\right) \end{array}$$ (7)

$$\begin{array}{l} e\cap f={\left(ef\right)}^{1-\gamma }{\left(e-ef\right)}^{\gamma } \end{array}$$ (8)

Here, λ, γ ∈ [0, 1] denote parameters. Based on these two operators, we can define the relevant operators of manifold granules, as follows: For ∀*x*_*i*_, *x*_*j*_ ∈ *X*, ∀*a*_*t*_ ∈ *A*, the operations between manifold granules induced by *x*_*i*_ and *x*_*j*_ can be realized by the following four operators.


$$\begin{array}{l} g_{x_{i}}\left(a_{t}\right)\cup g_{x_{j}}\left(a_{t}\right)=\int_{c_{k}\in C^{*}}s_{x_{i},c_{k}}\left(a_{t}\right)\cup s_{x_{j},c_{k}}\left(a_{t}\right) \end{array}$$ (9)

$$\begin{array}{l} g_{x_{i}}\left(a_{t}\right)\cap g_{x_{j}}\left(a_{t}\right)=\int_{c_{k}\in C^{*}}s_{x_{i},c_{k}}\left(a_{t}\right)\cap s_{x_{j},c_{k}}\left(a_{t}\right) \end{array}$$ (10)

$$\begin{array}{l} g_{x_{i}}\left(a_{t}\right)-g_{x_{j}}\left(a_{t}\right)=\int_{c_{k}\in C^{*}}s_{x_{i},c_{k}}\left(a_{t}\right)-s_{x_{j},c_{k}}\left(a_{t}\right) \end{array}$$ (11)

$$\begin{array}{l} g_{x_{i}}\left(a_{t}\right)⊕g_{x_{j}}\left(a_{t}\right)=g_{x_{i}}\left(a_{t}\right)\cup g_{x_{j}}\left(a_{t}\right)-g_{x_{i}}\left(a_{t}\right)\cap g_{x_{j}}\left(a_{t}\right) \end{array}$$ (12)

The distance between two fuzzy manifold granules can be defined as


$$\begin{array}{l} d\left(g_{x_{i}}\left(a_{t}\right),g_{x_{j}}\left(a_{t}\right)\right)=\frac{1}{\left|C^{*}\right|}\underset{c_{k}\in C^{*}}{\sum }\frac{\left|g_{x_{i}}\left(a_{t}\right)⊕g_{x_{j}}\left(a_{t}\right)\right|}{\left|g_{x_{i}}\left(a_{t}\right)\cup g_{x_{j}}\left(a_{t}\right)\right|} \end{array}$$ (13)

For ∀*A*_*t*_ ⊆ *A* and *A*_*t*_ = {*a*_*t*_1__, *a*_*t*_2__, ..., *a*_*t*_|_*A*__*t*_|__}, |*A*_*t*_| ≤ |*A*|, the fuzzy manifold granula vector induced by *x* on *A*_*t*_ can be defined as:


$$\begin{array}{l} \begin{array}{l} G_{x}\left(A_{t}\right)\\ =g_{x}\left(a_{t_{1}}\right)+g_{x}\left(a_{t_{2}}\right)+\cdot \cdot \cdot +g_{x}\left(a_{t_{\left|A_{t}\right|}}\right)\\ =\int_{a_{k}\in A_{t}}g_{x}\left(a_{k}\right)=\int_{a_{k}\in A_{t}}\int_{c_{j}\in C^{*}}s_{x_{i},c_{j}}\left(a_{k}\right) \end{array} \end{array}$$ (14)

Its module can be expressed as:


$$\begin{array}{l} \left|G_{x}\left(A_{t}\right)|=\underset{a_{k\in A_{t}}}{\sum }|g_{x}\left(a_{k}\right)\right| \end{array}$$ (15)

The operator of fuzzy manifold granula vector is given below.


$$\begin{array}{l} G_{x_{i}}\left(A_{t}\right)\cup G_{x_{j}}\left(A_{t}\right)=\int_{a_{k}\in A_{t}}g_{x_{i}}\left(a_{k}\right)\cup g_{x_{j}}\left(a_{k}\right) \end{array}$$ (16)

$$\begin{array}{l} G_{x_{i}}\left(A_{t}\right)\cap G_{x_{j}}\left(A_{t}\right)=\int_{a_{k}\in A_{t}}g_{x_{i}}\left(a_{k}\right)\cap g_{x_{j}}\left(a_{k}\right) \end{array}$$ (17)

$$\begin{array}{l} G_{x_{i}}\left(A_{t}\right)-G_{x_{j}}\left(A_{t}\right)=\int_{a_{k}\in A_{t}}g_{x_{i}}\left(a_{k}\right)-g_{x_{j}}\left(a_{k}\right) \end{array}$$ (18)

$$\begin{array}{l} G_{x_{i}}\left(A_{t}\right)⊕G_{x_{j}}\left(A_{t}\right)=\int_{a_{k}\in A_{t}}g_{x_{i}}\left(a_{k}\right)⊕g_{x_{j}}\left(a_{k}\right) \end{array}$$ (19)

The distance between two fuzzy manifold granula vectors can be defined as


$$\begin{array}{l} d\left(G_{x_{i}}\left(A_{t}\right),G_{x_{j}}\left(A_{t}\right)\right)=\frac{1}{\left|A_{t}|*|C^{*}\right|}\underset{a\in A_{t}}{\sum }\frac{\left|G_{x_{i}}\left(A_{t}\right)⊕G_{x_{j}}\left(A_{t}\right)\right|}{\left|G_{x_{i}}\left(A_{t}\right)\cup G_{x_{j}}\left(A_{t}\right)\right|} \end{array}$$ (20)

**Theorem 2**. For ∀*x*_*i*_, *x*_*j*_ ∈ *X*, ∀*A*_*t*_ ⊆ *A*, ∀*a*_*k*_ ∈ *A*_*t*_, the distance of the fuzzy manifold granula vector satisfies:


$$\begin{array}{l} 0\leq d\left(G_{x_{i}}\left(A_{t}\right),G_{x_{j}}\left(A_{t}\right)\right)\leq 1 \end{array}$$ (21)

Proof.Based on the definition of fuzzy manifold granules, it follows that $g_{x_{i}}\left(a_{k}\right)=\int_{c_{j}\in C^{*}}s_{x_{i},c_{j}}\left(a_{k}\right)$

$g_{x_{j}}\left(a_{k}\right)=\int_{c_{j}\in C^{*}}s_{x_{j},c_{j}}\left(a_{k}\right)$ From Equation 2, we have 0 ≤ *s*_*x*_*i*_, *c*_*j*__(*a*_*k*_) ≤ 1, 0 ≤ *s*_*x*_*j*_, *c*_*j*__(*a*_*k*_) ≤ 1. Due to $|g_{x_{i}}\left(a_{k}\right)|=\underset{c_{j}\in C^{*}}{\sum }s_{x_{i},c_{j}}\left(a_{k}\right)$, so $0\leq |g_{x_{i}}\left(a_{k}\right)|\leq |C^{*}|$, similarly $0\leq |g_{x_{j}}\left(a_{k}\right)|\leq |C^{*}|$.

Then define $G_{x_{i}}\left(A_{t}\right)=\underset{a_{t}\in T}{\int }\frac{g\left(x,a_{t}\right)}{a_{t}}$,$\left|G_{x_{i}}\left(A_{t}\right)|=\underset{a_{k\in A_{t}}}{\sum }|g_{x}\left(a_{k}\right)\right|$. Then $0\leq |G_{x_{i}}\left(A_{t}\right)|\leq |A_{t}|*|C^{*}|$. Similarly, $0\leq |G_{x_{j}}\left(A_{t}\right)|\leq |A_{t}|*|C^{*}|$. From $0\leq \underset{a\in T}{\sum }\frac{\left|G_{x_{i}}\left(A_{t}\right)⊕G_{x_{j}}\left(A_{t}\right)\right|}{\left|G_{x_{i}}\left(A_{t}\right)\cup G_{x_{j}}\left(A_{t}\right)\right|}\leq |A_{t}|*|C^{*}|$, Divide both sides by $\left|A_{t}|*|C^{*}\right|$ to get

$0\leq \frac{1}{\left|A_{t}|*|C^{*}\right|}\underset{a\in T}{\sum }\frac{\left|G_{x_{i}}\left(A_{t}\right)⊕G_{x_{j}}\left(A_{t}\right)\right|}{\left|G_{x_{i}}\left(A_{t}\right)\cup G_{x_{j}}\left(A_{t}\right)\right|}\leq 1$, that is, 0 ≤ *d*(*G*_*x*_*i*__(*A*_*t*_), *G*_*x*_*j*__(*A*_*t*_)) ≤ 1.

**Theorem 3 (monotonicity)**. For any *x* ∈ *X*, the attribute subsets *B*_*t*_ and *A*_*t*_ satisfy *B*_*t*_ ⊆ *A*_*t*_ ⊆ *A*. Let *G*_*x*_*i*__(*A*_*t*_) and *G*_*x*_*i*__(*B*_*t*_) denote the fuzzy manifold granule vectors of sample *x*_*i*_ corresponding to attribute subsets *A*_*t*_ and *B*_*t*_, respectively. Then |*G*_*x*_(*B*_*t*_)| ≤ |*G*_*x*_(*A*_*t*_)| holds.

*Proof*: For ∀*a*_*t*_ ∈ *B*_*t*_, $G_{x}\left(B_{t}\right)=\int_{t=1}^{\left|B_{t}\right|}g_{x}\left(a_{t}\right)$, because *B*_*t*_ ⊆ *A*_*t*_, so *a*_*t*_ ∈ *A*_*t*_, $G_{x}\left(A_{t}\right)=\int_{t=1}^{\left|A_{t}\right|}g_{x}\left(a_{t}\right)$, because *B*_*t*_⊆*A*_*t*_⊆*A*, so for any *a* ∈ *B*_*t*_, then there must be *a* ∈ *A*_*t*_ and satisfy |*B*_*t*_| ≤ |*A*_*t*_|, that is, if *g*_*x*_(*a*) ∈ *G*_*x*_(*B*_*t*_), then there is *g*_*x*_(*a*) ∈ *G*_*x*_(*A*_*t*_). In summary, the inequality |*G*_*x*_(*B*_*t*_)| ≤ |*G*_*x*_(*A*_*t*_)| holds.

The parallel granulation process based on Dask is detailed in Algorithm 2.

Algorithm 2 Parallel fuzzy manifold granulation using dask. 
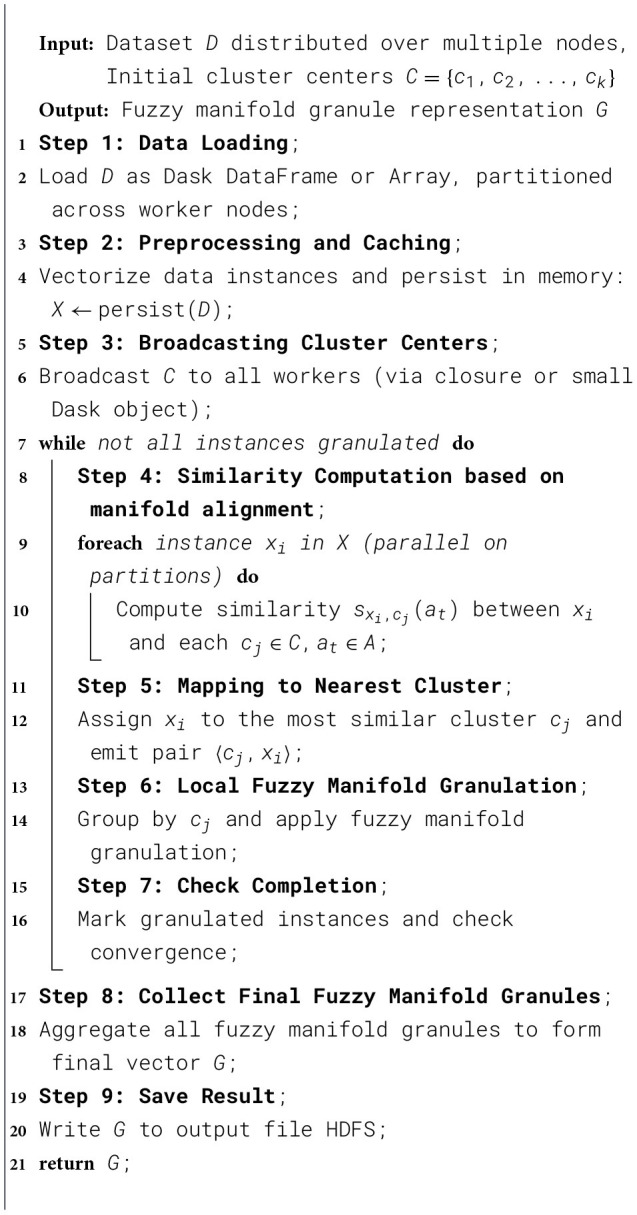

## 4 Fuzzy manifold granule hypersurface classifier

Assuming a dataset of *n* nonlinear samples, there exist infinitely many decision boundaries in the feature space that achieve zero classification error. The key question is: among these, which decision surface is optimal? To address this, we map the problem into the fuzzy manifold granule space. Leveraging the atomic properties inherent in this space, the classification system represents each sample as a fuzzy manifold granule. Then a fuzzy manifold granula vector is created from the fuzzy manifold granule. In this way, the category and the fuzzy manifold granula vector form a fuzzy manifold granula vector sample set. To identify the desired fuzzy manifold granule hypersurface, we introduce parameters ω and *b* to formulate its defining equation. These parameters, ω and *b*, are determined based on the training samples. Therefore, this classification process can be transformed into finding fuzzy manifold granula vectors ω and *b* in fuzzy manifold granula space. Based on the atomic attributes, the classification system maps each sample into a fuzzy manifold granule, thereby constructing a fuzzy manifold granule sample set composed of these granules. The decision equation calculates the category. The calculation process is as follows: First, the fuzzy manifold granulation sample; next, the problem is transformed To solve the equation, we need to find the fuzzy manifold granula vectors ω and *b*. The fuzzy manifold granule hypersurface equation is established, and the optimal parameters ω and *b* are obtained by minimizing the loss function to maximize the margin of the hypersurface. Before proceeding with the problem solution, we assume that the prediction error, defined as the difference between the predicted and true values, follows a normal distribution and arises from a linear classification model. The parameters ω and *b* define the fuzzy manifold granule hypersurface that maximizes the margin. The optimization process is carried out within the fuzzy manifold granule space using the particle swarm optimization algorithm. The process described above is formalized as:

**Definition 1**. Assume a classification system 𝕊 = (*X, Y, A, V*, τ), where *X* = {*x*_1_, *x*_2_, …, *x*_*n*_} is the sample set with each sample $x_{i}\in {\mathbb{R}}^{m}$. The label set is given by *Y* = {*y*_1_, *y*_2_, …, *y*_*n*_}, where each label *y*_*i*_ ∈ {0, 1, …, *l*} corresponds to the sample *x*_*i*_. The attribute set *A* = {*a*_1_, *a*_2_, …, *a*_*m*_} is a non-empty finite set, and *V* = ⋃_*a* ∈ *A*_ *V*_*a*_ denotes the union of attribute value ranges, where *V*_*a*_ is the domain of attribute *a*. The information function τ: *X* × *A* → *V* assigns a value to each attribute of each object; that is, for all *a* ∈ *A* and *x* ∈ *X*, τ(*x, a*) ∈ *V*_*a*_.

Each sample is represented by a fuzzy manifold granula vector *G*_*x*_*i*__(*A*) along with its category *y*_*i*_, forming pairs 〈*G*_*x*_*i*__(*A*), *y*_*i*_〉 for *i* = 1, 2, …, *n*. The fuzzy manifold granula vector sample set is defined as


$$G\left(X,A,Y\right)=\left\{\langle G_{x_{i}}\left(A\right),y_{i}\rangle |x_{i}\in X\right\}.$$

Within the fuzzy manifold granula space, the hypersurface equation is defined by the following optimization problem:


$$\begin{array}{l} L\left(\omega ,b\right)=\underset{\omega ,b}{\min}\left\{\underset{x_{i}\in X}{\sum }{\left(\log|\omega \wedge G_{x_{i}}\left(A\right)\vee b|-y_{i}\right)}^{2}+\lambda ∥\omega {∥}^{2}\right\}, \end{array}$$ (22)

where ω and *b* are fuzzy manifold granule vectors, and λ ∈ ℝ is a regularization parameter.

Let ω^*^ and b^*^ be the optimal solutions of the above hypersurface equations, then the classification decision function can be written as


$$\begin{array}{l} f\left(x\right)=\underset{0\leq y\leq l}{\arg\min}\left\{\left|log|\omega^{*}\wedge G_{x}\left(A\right)\vee b^{*}|-y\right|\right\} \end{array}$$ (23)

The detailed mathematical framework and property definitions are systemically formulated in Equations 3–22.

### 4.1 Solving parameters

We employ the particle swarm algorithm to solve Equation 23. The particle swarm algorithm maps “particles” to the problem to be solved, assumptions and constraints. They follow the same pathfinding rules as the swarm, that is, the particles of the swarm have both speed and direction. During each iteration, a particle updates its velocity and position based on both its personal best experience and the swarm's collective knowledge. The individual best—also called the personal best—is the historically optimal position found by the particle itself, while the global best represents the historically optimal position discovered by the entire swarm. As a model governed by velocity-position update equations, the particle swarm optimization (PSO) algorithm enables each particle not only to independently search for an optimal solution but also to share and learn from the experiences of others, thereby enhancing overall convergence.

In the context of the fuzzy manifold granule space, each particle freely explores the search space, continually updating its personal best. Through communication among particles, the global best is identified as the best solution found so far across the swarm. The velocity and position of each particle are iteratively adjusted using both the particle's own personal best and the global best known to the swarm.

The iterative update process can be summarized as follows:

**Step 1**. Initialize a set of fuzzy manifold granule vectors γ_1_, γ_2_, …, γ_*N*_ randomly, where each particle is denoted as γ_*i*_ = (ω_*i*_, *b*_*i*_), along with their associated velocities *v*_1_, *v*_2_, …, *v*_*N*_. Set the iteration counter *I* = 0 and specify the maximum number of iterations *MaxI*.

**Step 2**. Compute the loss value *L*(γ_*i*_) for each particle and assign their personal best positions as *pB*_*i*_ = γ_*i*_, for *i* = 1, 2, …, *N*.

**Step 3**. Identify the global best position as


$$gB=\arg\underset{1\leq j\leq N}{\min}L\left(pB_{j}\right)$$

**Step 4**. Update the velocity and position of each particle based on its personal best and the global best positions using:


$$\begin{array}{l} v_{i}\leftarrow r_{0}\cdot v_{i}+c_{1}\cdot r_{1}\cdot \left(pB_{i}-\gamma_{i}\right)+c_{2}\cdot r_{2}\cdot \left(gB-\gamma_{i}\right) \end{array}$$ (24)

$$\begin{array}{l} \gamma_{i}^{*}\leftarrow \gamma_{i} \end{array}$$ (25)

$$\begin{array}{l} \gamma_{i}\leftarrow \gamma_{i}+v_{i} \end{array}$$ (26)

**Step 5**. Evaluate the fitness of the updated particle; if $L\left(\gamma_{i}^{*}\right)>L\left(\gamma_{i}\right)$, update the personal best position as *pB*_*i*_ = γ_*i*_, otherwise retain $pB_{i}=\gamma_{i}^{*}$.

**Step 6**. Increment the iteration counter: *I* ← *I* + 1.

**Step 7**. If *I* > *MaxI*, proceed to Step 8; otherwise, return to Step 2 for the next iteration.

**Step 8**. Output the global best solution *gB* as the final result.

Algorithm 3 shows the pseudo code for solving the parameters. The precise derivation and update rules for the proposed model are expressed in Equations 24–26.

Algorithm 3 Parameters solving algorithm for fuzzy manifold granule hypersurface classifier. 
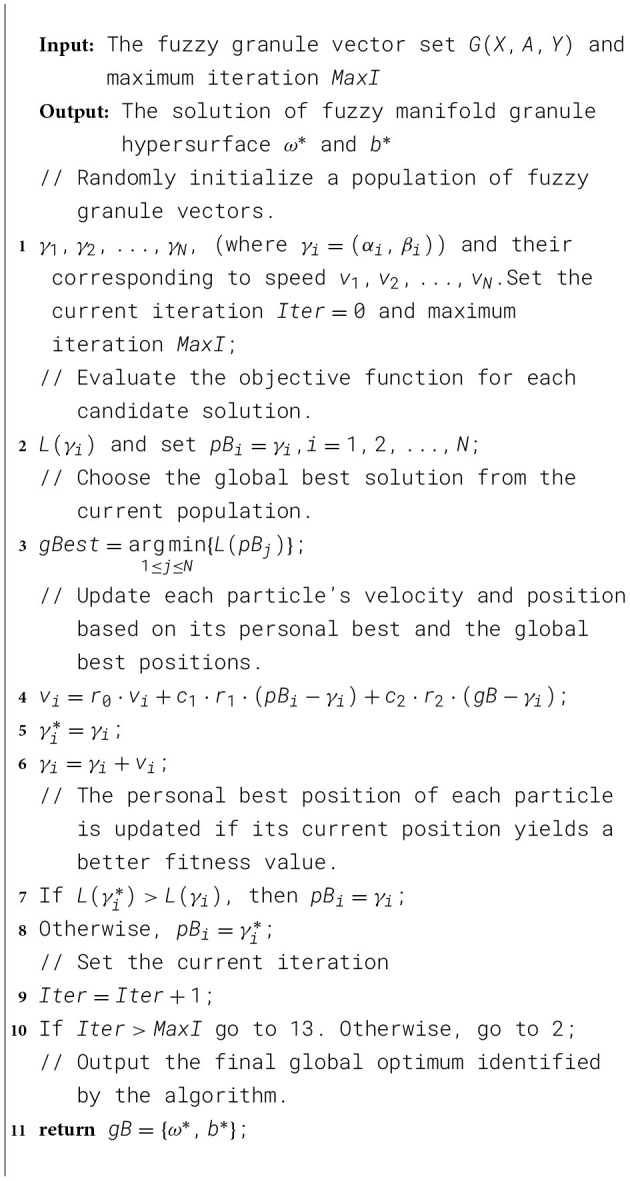

## 5 Boosted fuzzy manifold granule hypersurface classification model

To further improve the performance of the algorithm, based on the previous work, we propose a decision model that integrates multiple fuzzy manifold granule models. It constructs a strong fuzzy manifold particle classification model by combining multiple weak fuzzy manifold particle classification models. The basic principle is: each time a new fuzzy manifold particle classification model is trained, a higher weight is given to the previously misclassified fuzzy manifold particle samples, so that the new fuzzy manifold granule classifier pays more attention to these difficult-to-classify samples. The principle is as follows.

**Step 1**. Compute the similarity between samples via the local topology-preserving metric (see Equation 1).

**Step 2**. Calculate the sample cluster center using Global Optimization Clustering Algorithm with Local Topology Structure Preservation.

**Step 3**. Randomly divide the sample set into several sample subsets.

**Step 4**. Use parallel method to fuzzy granulate each sub-sample set to obtain the full set of fuzzy manifold granule vectors.

**Step 5**. Obtain the fuzzy manifold particle vector subset based on the sample and cluster center.

**Step 6**. Initialize weights.

**Step 7**. Cycle training *t* = 1, 2, ..., *T* rounds to get *T* basis fuzzy manifold granule classifiers.

**Step 8**. Final output of boosted fuzzy manifold granule classifier: The final boosted fuzzy manifold granule classifier is a weighted combination of each basis fuzzy manifold granule classifier.

The BFMGHC gradually improves classification performance by continuously adjusting the weights of the samples and the weak fuzzy manifold granule hypersurface classifier. Its advantage is that it can significantly improve the performance of weak classifiers while being less prone to overfitting problems, especially in the case of weak fuzzy manifold granule hypersurface classifiers and good data quality. Algorithm 4 describes the detailed process.

Algorithm 4 BFMGHC algorithm. 
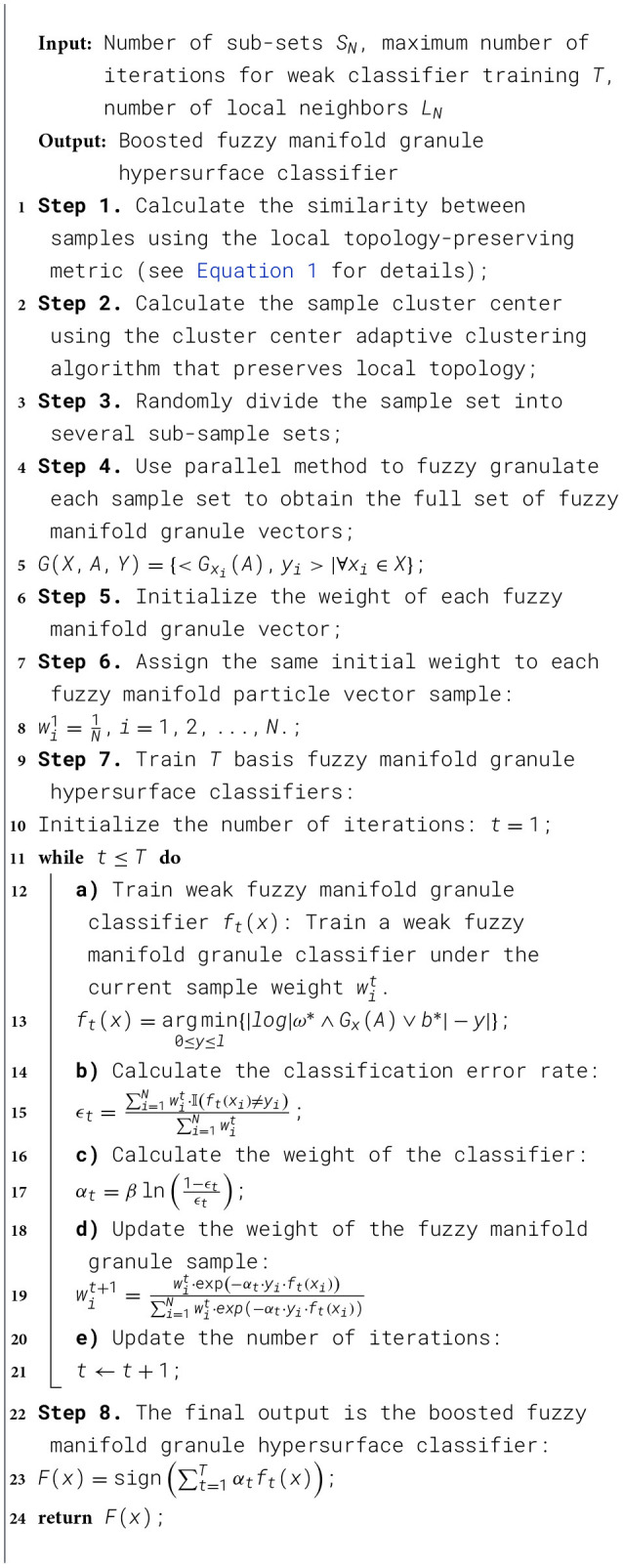

After solving (training) the model parameters, we can get the fuzzy manifold particle vector set, cluster center set C^*^ and other results. For a given sample *x*, calculate its fuzzy manifold particle vector *G*_*x*_(*A*), and calculate the classification result according to $F\left(x\right)=\mathrm{sign}\left(\overset{T}{\underset{t=1}{\sum }}\alpha_{t}f_{t}\left(x\right)\right)$ (see Algorithm 5 for details).

Algorithm 5 Prediction. 
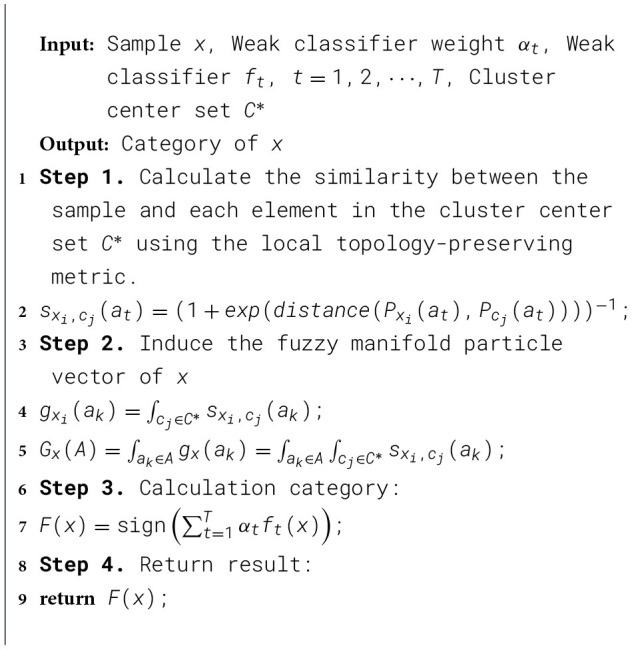

## 6 Experimental validation

This section presents a rigorous evaluation of BFMGHC. The experiments are structured in four parts: a description of the experimental setup and datasets, a comparative study against state-of-the-art algorithms on the datasets, and a comprehensive discussion of the results and implications.

### 6.1 Experimental environment and dataset

All experiments were conducted on a MacBook Pro (Model: MX2W3CH/A) equipped with an Apple M4 Max chip, comprising a 16-core CPU and a 40-core GPU, along with 48GB of memory. The implementation was carried out in Python using Conda and VSCode as the development environment. The data sets used in our experiments were obtained from the UCI Machine Learning Repository. To ensure the robustness of the experimental results and eliminate potential biases from data partitioning, all experiments are conducted using a standard 10-fold cross-validation protocol.

### 6.2 Algorithmic benchmarking

To assess the performance of BFMGHC, we compared it with five widely recognized machine learning models. XGBoost (Chen and Guestrin, 2016), LightGBM (Ke et al., 2017), HistGradientBoosting (Pedregosa et al., 2011), Random Forest (Breiman, 2001), and Support Vector Machines (SVM) (Vapnik, 1995). Evaluation was performed on three benchmark datasets: Bank Marketing, Default of Credit Card Clients, and German Credit Data, all of which are standard in financial risk modeling.

**Figure 2:**
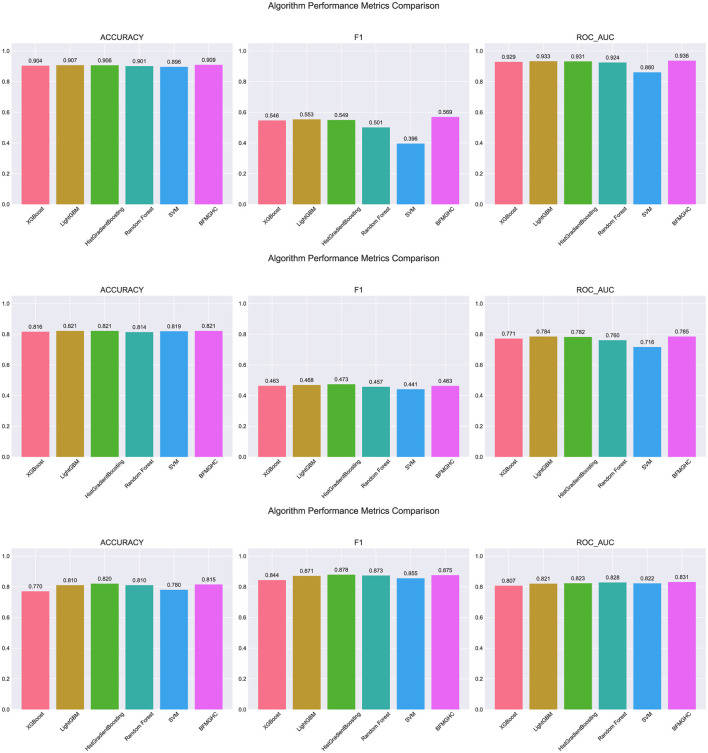
The comparison of accuracy, F1 score, and ROC_AUC of algorithms.

Figure 2 (top) demonstrates the effectiveness of different models when applied to the Bank Marketing dataset. Given the inherently imbalanced nature of financial datasets, traditional metrics such as accuracy may not reveal true predictive capabilities. Therefore, we emphasize the ROC AUC, which provides a threshold-independent assessment of the model discrimination power. As indicated by the results, XGBoost and LightGBM produced ROC AUCs of 0.9287 and 0.9326, with F1 scores of 0.5464 and 0.5533, respectively. Random Forest and HistGradientBoosting followed closely, while SVM performed poorly with a ROC AUC of 0.8605. The BFMGHC model outperformed all baselines, achieving an accuracy of 0.9091, an F1 score of 0.5692, and a leading ROC AUC of 0.9362. Figure 2 (middle) details the results on the Default of Credit Card Clients dataset, which exhibits even more severe class imbalance. Although LightGBM and HistGradientBoosting both achieved high accuracy (0.822), their ROC AUCs remained around 0.782, slightly ahead of XGBoost (0.7706), and Random Forest (0.7582). SVM again performed poorly. BFMGHC achieved the highest ROC AUC of 0.7850, underscoring its superior ability to detect rare default events.

Figure 2 (bottom) evaluates the models on the German Credit Data dataset. Once again, BFMGHC exhibited the strongest discriminative capacity, achieving a ROC AUC of 0.8309, outperforming all baselines. Despite other models achieving similar levels of accuracy and F1 score, they were unable to effectively handle class imbalance, reinforcing the critical importance of ROC AUC in financial prediction tasks.

### 6.3 Training efficiency and accuracy analysis

**Figure 3:**
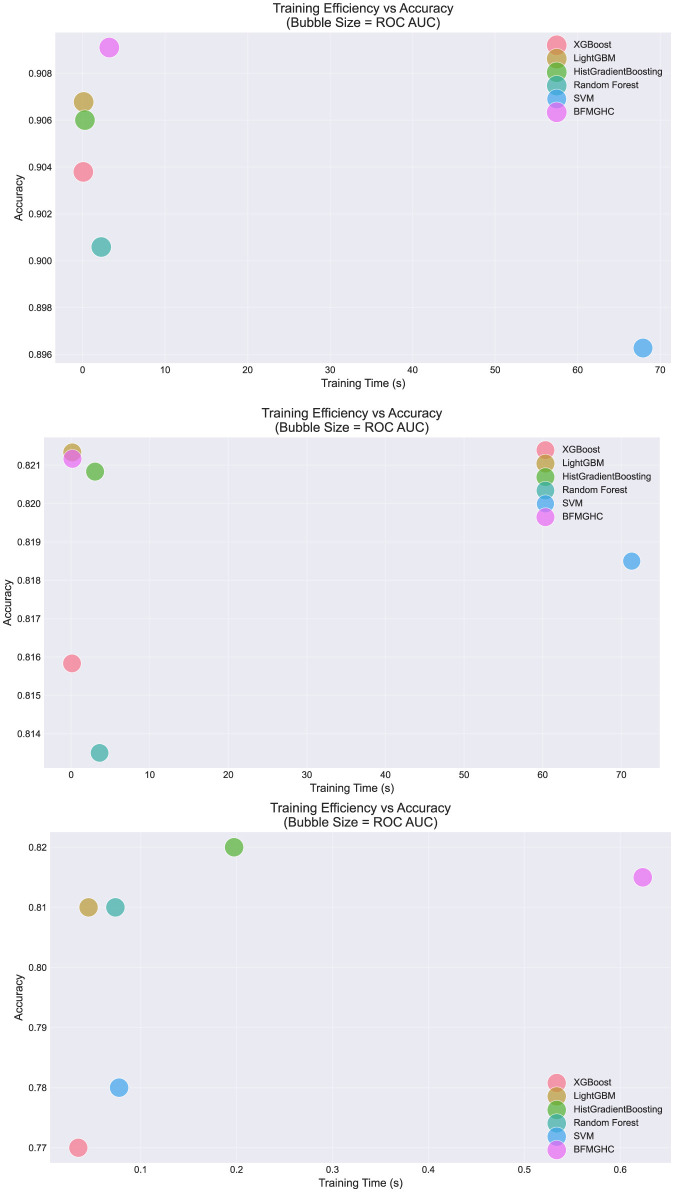
The comparison of the efficiency and the accuracy of algorithms.

Figure 3 compares training efficiency and final accuracy across the three datasets. These charts illustrate the balance between computational cost and predictive accuracy for each model. Although some models exhibit faster training times, they often compromise accuracy. BFMGHC achieves a favorable trade-off, demonstrating competitive training speed without sacrificing performance.

### 6.4 Confusion matrix evaluation

**Figure 4:**
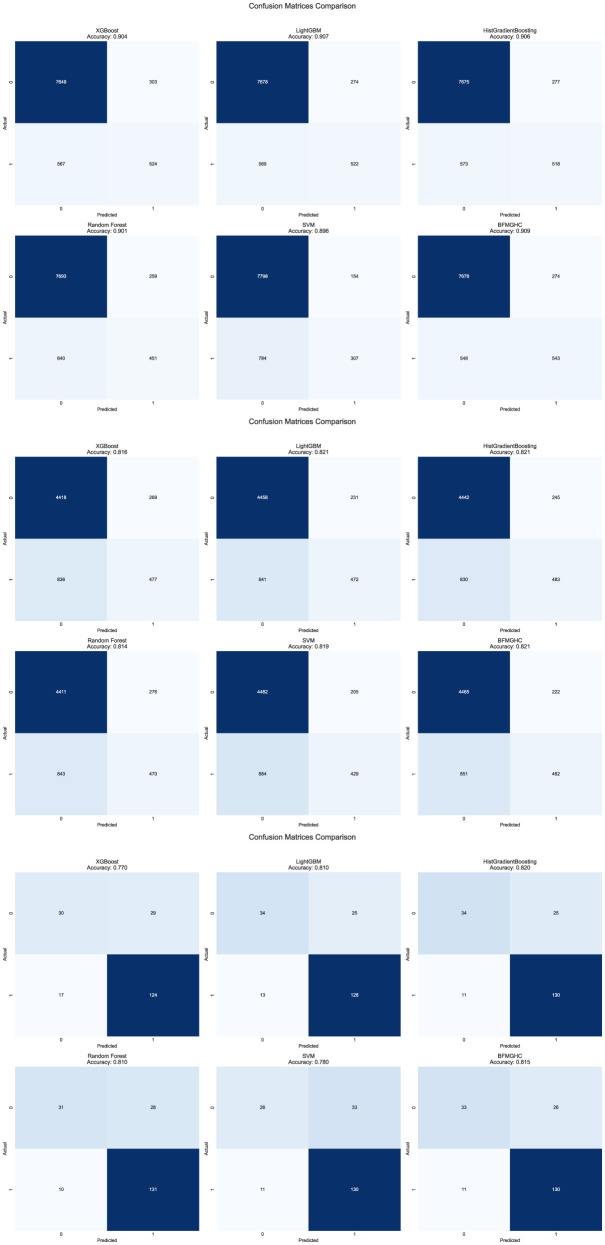
The comparison of confusion matrices of the algorithms.

Figure 4 presents the confusion matrices for all models across the datasets. These matrices provide insight into the models' accurate positive and false negative rates, which are crucial in financial contexts where missing a potential defaulter can be costly. BFMGHC consistently demonstrates fewer false negatives, highlighting its robustness in class-imbalanced scenarios.

### 6.5 Multi-metric radar visualization

**Figure 5:**
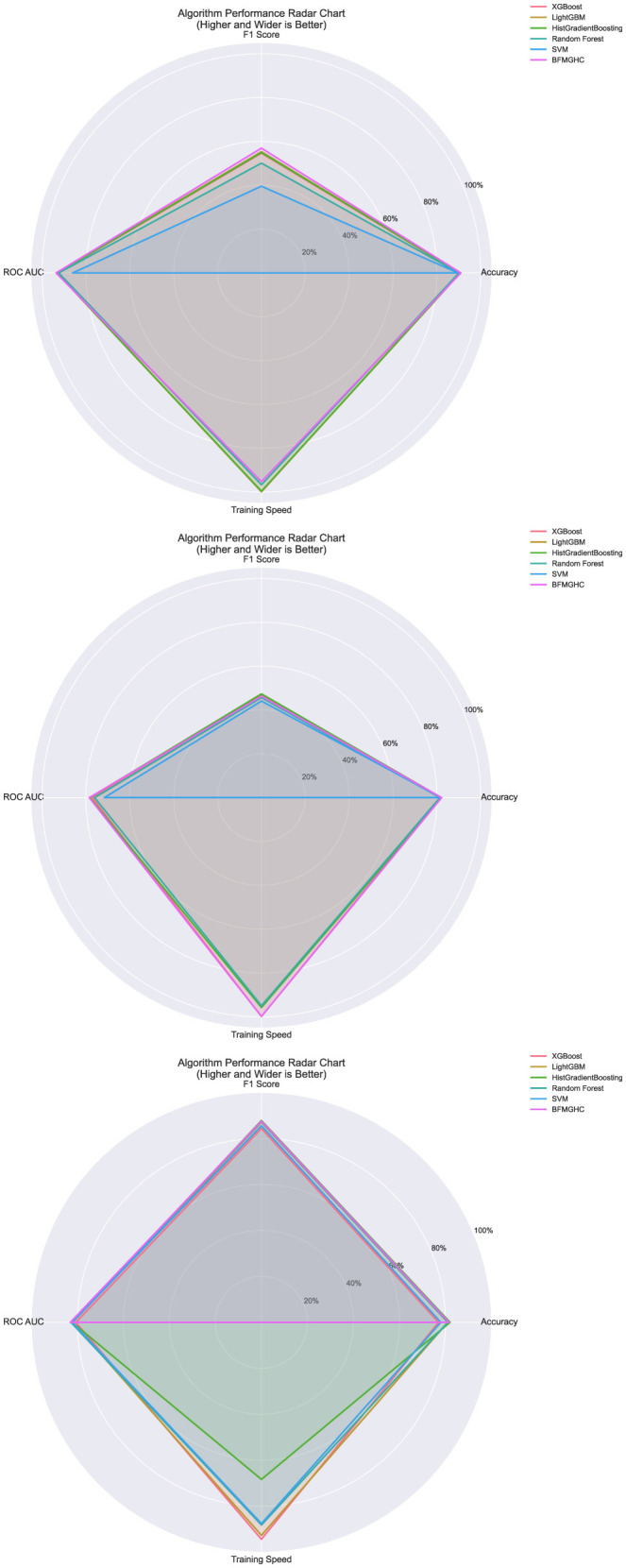
Comprehensive indicator radar chart of the algorithms.

Figure 5 illustrates the comparative performance of models using radar charts based on four essential metrics: Accuracy, F1 Score, Training Time, and ROC AUC. The radar plots deliver an integrated overview of the models' behavior across multiple evaluation criteria. BFMGHC exhibits the most balanced and dominant profile, particularly excelling in ROC AUC—a metric vital for applications in financial risk assessment.

### 6.6 Summary

The BFMGHC model consistently outperforms leading machine learning models in key financial classification tasks, particularly in the ROC AUC metric. This indicates its strong generalization ability and practical utility in identifying high-risk clients in imbalanced financial datasets. The superior performance in ROC AUC validates its applicability for deployment in real-world financial risk assessment systems, offering enhanced accuracy and interpretability for institutions seeking reliable predictive analytics tools.

## 7 Discussion

Although the BFMGHC algorithm is validated on financial risk assessment datasets in this study, its underlying architecture—characterized by low-dimensional manifold tracking, fuzzy cognitive granulation, and interpretable ensemble decision-making—holds profound potential for adaptive control systems and autonomous robotics.

In real-world robotic control, default risks and market volatility are mathematically analogous to environmental disturbances and sensory uncertainties. The capability of BFMGHC to preserve local topological structures allows an autonomous agent to maintain stable and interpretable behavior when interacting with dynamic environments.

The BFMGHC proposed in this study has achieved innovations and breakthroughs in multiple levels of intelligent modeling methods. Compared with traditional point or sample-based classification methods, this paper introduces fuzziness and manifold structure from the perspective of “information granules,” showing higher robustness and interpretability when processing complex, high-dimensional, and uncertain data.

First, we introduce a manifold-preserving local topological similarity measurement method in metric modeling, which breaks away from the limitation of the “Euclidean distance” due to its inadequate ability to express complex spaces. By constructing a sample-centered local popular topological graph, we effectively capture the structural relationship of the potential data manifold, and on this basis propose a globally optimized clustering method. Combined with the distributed implementation of the Dask parallel framework, this method is also scalable and suitable for large-scale data scenarios.

Secondly, we introduce a fuzzy hypersurface classification model at the granular level in the fuzzy manifold granule space generated by clustering, and optimize the classifier's parameters through the particle swarm algorithm, effectively realizing the coordinated adaptation of the model structure and parameters. In this process, the fuzzy expression between particles enables the model to handle boundary samples and transition areas more naturally, thereby enhancing the model's stability under fuzzy boundaries and noisy samples.

Furthermore, we designed an integrated framework to weightedly integrate multiple basis fuzzy manifold particle hypersurface classifiers, while maintaining model diversity, improving the overall prediction performance and generalization ability. By assigning weights to different particles and classifiers, this framework reflects a certain degree of explanatory power and controllability, providing a traceable basis for the decision-making process.

The effectiveness of the approach was empirically demonstrated using three real datasets pertaining to financial risk assessment. The results confirm that the model proposed in this study delivers superior performance with respect to accuracy, robustness, and interpretability, especially under conditions involving nonlinear data distributions and fuzzy classification boundaries. Compared to traditional methods, it offers significant advantages.

It is worth noting that, although the model in this paper demonstrates good performance, there is still room for further optimization. For example, the construction of particle space and manifold approximation still rely on the construction and density estimation of the neighborhood graph. In future studies, incorporating self-supervised representation learning and geometric deep learning could further improve the expressive power of particles. In addition, the integrated weight mechanism of fuzzy particles still relies heavily on heuristic methods. In the future, it may be possible to explore the introduction of a game theory or reinforcement learning framework to achieve dynamic weight adjustment.

In summary, the methodology introduced in this study demonstrates strong potential in theoretical design, system implementation, and practical application, providing a beneficial path for the next generation of interpretable, highly robust, and uncertainty-friendly intelligent classification models.

## Data Availability

Publicly available datasets were analyzed in this study. This data can be found here: https://archive.ics.uci.edu/datasets/?search=Default+of+Credit+Card+Clients+, https://archive.ics.uci.edu/datasets/?search=Bank+Marketing, and https://archive.ics.uci.edu/dataset/144/statlog+german+credit+data.
